# Trends in bacteremic infection due to Streptococcus pyogenes (group A streptococcus), 1986-1995.

**DOI:** 10.3201/eid0201.960107

**Published:** 1996

**Authors:** D M Musher, R J Hamill, C E Wright, J E Clarridge, C M Ashton

**Affiliations:** Veterans Affairs Medical Center, Houston Veterans Affairs Health Services Research and Development Field Program, Texas, USA.


### Trends in Bacteremic Infection Due to *Streptococcus pyogenes* (Group A Streptococcus), 1986-1995

During the past 7 years, severe invasive infections caused by *Streptococcus pyogenes* (group A streptococcus [GAS]) have been reported with increasing frequency (1). It is not certain whether these reports reflect actual increase in the incidence of disease caused by this pathogenic bacterium or merely enhanced awareness and interest on the part of the medical community. This difficulty prevails whether the apparent resurgence of life-threatening infection is described within a region (2), a province (3), or in discrete outbreaks (4,5) and is heightened by the fact that streptococcal infection has not been a reportable disease in most locales.

An outbreak of infection may awaken interest in streptococcal disease, leading to publication bias (6,7). Continued study may show that what appears to be a general increase in the incidence of GAS infection may actually represent an outbreak, as has been demonstrated in Colorado (8,9), Sweden (10), and the United Kingdom (11). In a laboratory-based study, Burkert and Watanakunakorn (12) found that the frequency and apparent severity of bacteremic infection due to *S. pyogenes* did not change from 1980 to 1989. A meticulous, population-based study in Pima County, Arizona (13), found no change in the incidence of invasive GAS disease between 1985 and 1990 but did suggest the emergence of streptococcal toxic shock syndrome in the late 1980s, on the basis of the appearance of six such cases in 1987-1990 compared to zero cases in 1985-1987. In contrast, a preliminary, population-based report from Israel (14) found no such increase.

The Houston Veterans Affairs Medical Center (HVAMC) serves about 50,000 eligible veterans, most in Harris County, Texas, and surrounding counties, although a small number of these veterans live elsewhere in Texas or in bordering states. Most patients are middle-aged or elderly men, although approximately 20% are under the age of 40. Within the past decade, there have been broader medical options for some of our patients, especially those who are indigent and/or elderly, and narrower options for others, especially those whose medical insurance was initially marginal. Nevertheless, most persons who select the HVAMC for care of any problem tend to identify themselves with this medical center and look to it to provide complete medical care (15).

In contrast to that reported by some medical centers in the United States, our clinical experience did not suggest that the number or severity of infections due to *S. pyogenes* had increased during the past decade. Because our population was relatively stable and data were available in our Microbiology Laboratory for 1986 through 1995, we studied trends in bacteremic infection at the medical center during this period.

In the 9 years under study, the number of yearly inpatient admissions to the HVAMC decreased by 4.1%, while the number of outpatient visits increased by 33.9%. The number of patients who, in a given year, sought medical attention at the center (determined by social security numbers), increased by 18.7%, with a 5.2% decrease in those persons admitted to the hospital and a 19.4% increase in those who sought outpatient care. During this period, the number of blood specimens submitted for cultures each year remained essentially unchanged. We have noted no change in the clinical findings that prompt interns and residents to request blood cultures, although the possibility of unrecognized, subtle changes cannot be excluded.

The rate of isolation of GAS from blood cultures (Figure 1) or from all sterile sites (data not shown) remained unchanged between 1986 and 1995. The number of positive blood cultures tended to be lower in the summer and early fall and to rise in midwinter, but the average number has remained at approximately five to eight per year during the entire decade. The frequency with which GAS were isolated from all specimens declined significantly ($p = 0.003$) during the decade under observation.

These data suggest that the frequency of bacteremic infection due to *S. pyogenes* has not increased in a population of veterans residing in and around Houston, Texas. The number of isolates of *S. pyogenes* from blood and from all sterile sites has remained unchanged, whereas the number of persons served in inpatient and outpatient encounters actually increased. The slight, but significant decrease in the total number of isolates from all sources might suggest that an

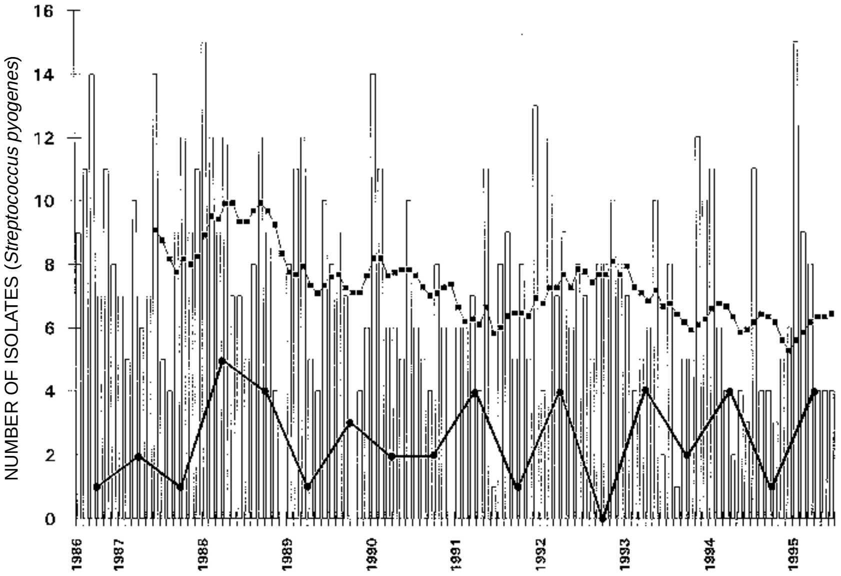

Figure 1. The bars show the number of isolates of *Streptococcus pyogenes* (GAS) each month at the VAMC, Houston. The upper line connecting solid squares indicates the running monthly average (average of the preceding 12 months). The lower line connecting solid circles indicates the number of blood cultures positive for *S. pyogenes* during each 6-month period.

unchanging number of blood isolates represents an increased prevalence of virulent strains, but the large number of variables involved in collecting all specimens makes these data difficult to interpret.

By showing that the incidence of *S. pyogenes* bacteremia has not changed, these results appear to support our anecdotal observation that the incidence of life-threatening GAS infection has not increased. Without reviewing every single case, which is best done prospectively rather than retrospectively, we cannot exclude the possibility that the number of cases associated with streptococcal shock syndrome has increased, as was suggested in Pima County (13). Even that study, however, showed only six cases of shock (two per year) in 1987 through 1990 compared to none in 1985 through 1987; one wonders whether bias in recording data might have been partially responsible, and whether this increase will persist. We also do not know what proportion of our cases is associated with necrotizing fasciitis, although it must be noted that Hoge, et al. (13) specifically did not document the association between this clinical syndrome and streptococcal toxic shock. Our findings cannot exclude the possibility that serious GAS has increased in younger adults, as has been reported by others (1,13), since our population tends to be (but is not exclusively) middle-aged or older. Also, the effect of increases may not have been observed in Houston. In any case, reporting bias is difficult to avoid, and prospective population-based studies must be carried out over an adequate number of years before they can provide valid conclusions.

If the frequency of severe GAS infection has, in fact, increased, a virulent clone or the mutation of a clone that has antigens to which most adults lack antibody could be responsible. Serotype M1 has predominated in reports of streptococcal toxic shock syndrome (16-18). Some investigators (19) have proposed that allelic variation in the streptococcal pyrogenic exotoxin A gene (speA) may contribute to disease severity; however, others have not found a high prevalence of isolates that produce pyrogenic exotoxin A among organisms that cause streptococcal toxic shock syndrome. Although the gene that encodes pyrogenic exotoxin B, a cysteine protease, has been universally present in virulent isolates, this gene is also present in nearly all other GAS isolates; therefore, its importance in toxic shock-producing strains remains unclear. Finally, changes in herd immunity to certain exotoxin B variants and/or M protein serotypes may result in an increased susceptibility to newly emerging mutant strains (20).

### Acknowledgment

The authors are grateful to Nancy J. Petersen, M.S., for help in obtaining VA Medical Center utilization data.

**Daniel M. Musher, Richard J. Hamill, Charles E. Wright, Jill E. Clarridge, Carol M. Ashton**
Veterans Affairs Medical Center, Houston Veterans Affairs Health Services Research and Development Field Program, and Baylor College of Medicine, Houston, Texas

### References

1. Stevens DL. Streptococcal toxic shock syndrome: spectrum of disease, pathogenesis, and new concepts in treatment. Emerging Infectious Diseases 1995; 1:69-78.
2. Stevens DL, Tanner MH, Winship J, et. al. Reappearance of scarlet fever toxin A among streptococci in the Rocky Mountain West: severe group A streptococcal infections associated with a toxic shock-like syndrome. N Engl J Med 1989; 321:1-7.
3. McGeer K, Green R, Cann D, et. al. Changing epidemiology of invasive group A streptococcal infection—population based surveillance, Ontario Canada, 1992-1995. Presented at the 35th Interscience Conference on Antimicrobial Agents and Chemotherapy, 1995; Abstract K135,
4. Cockerill F III, Thompson R, Roberson F, et. al. Outbreak of invasive group A streptococcal infections (ISI) in residents of southeastern Minnesota. Presented at the 35th Interscience Conference on Antimicrobial Agents and Chemotherapy, 1995; Abstract K137.
5. Levine OS, Turf E, Ginsberg R, et. al. An outbreak of invasive group A streptococcal disease in the Shenandoah Valley of Virginia. Presented at the 35th Interscience Conference on Antimicrobial Agents and Chemotherapy, 1995; Abstract K134.
6. Braunstein H. Characteristics of group A streptococcal bacteremia in patients at the San Bernardino County Medical Center. Rev Infect Dis 1991;13:8-11.
7. Demers B, Simor AE, Vellend H, et al. Severe invasive group A streptococcal infections in Ontario, Canada: 1987–1991. Clin Infect Dis 1993;16:792-800.
8. Centers for Disease Control. Group A beta-hemolytic streptococcal bacteremia—Colorado, 1989. MMWR 1990;39:3-11.
9. Colorado Department of Health. Surveillance for group A streptococcal bacteremia in metropolitan Denver. Colo Dis Bull 1991;2:18.
10. Stromberg A, Romanus V, Burman LG. Outbreak of group A streptococcal bacteremia in Sweden: an epidemiologic and clinical study. J Infect Dis 1991;164:595-8.
11. Centers for Disease Control and Prevention. Invasive group A streptococcal infections—United Kingdom, 1994. MMWR 1994;43:401-2.
12. Burkert T, Watanakunakorn C. Group A streptococcal bacteremia in a community teaching hospital—1980–1989. Clin Infect Dis 1992;14:29-37.
13. Hoge CW, Schwartz B, Talkington DF, et al. The changing epidemiology of invasive group A streptococcal infections and the emergence of streptococcal toxic shock-like syndrome. JAMA 1993;269:384-9.
14. Ashkenazi S, Livni G, Leibovici L, et. al. Trends in incidence and severity of group A streptococcal (GAS) bacteremia. Presented at the 35th Interscience Conference on Antimicrobial Agents and Chemotherapy, 1995; Abstract K138.
15. Fleming C, Fisher E, Chang C, et. al. Studying outcomes and hospital utilization in the elderly. Med Care 1992; 30:377-91.
16. Holm SE, Norrby A, Bergholm A-M, Norgren M. Aspects of pathogenesis of serious group A streptococcal infections in Sweden, 1988–1989. J Infect Dis 1992;166:31-7.
17. Cleary PP, Kaplan EL, Handley JP, et al. Clonal basis for resurgence of serious *Streptococcus pyogenes* disease in the 1980s. Lancet 1992;339:518-21.
18. Talkington DF, Schwartz B, Black CM, et al. Association of phenotypic and genotypic characteristics of invasive *Streptococcus pyogenes* isolates with clinical components of streptococcal toxic shock syndrome. Infect Immun 1993;61:3369-74.
19. Musser JM, Kapur V, Kanjilal S, et al. Geographic and temporal distribution and molecular characterization of two highly pathogenic clones of *Streptococcus pyogenes* expression allelic variants of pyrogenic exotoxin A (scarlet fever toxin). J Infect Dis 1993;167:337-46.
20. Musser JM, Nelson K, Selander RK, et al. Temporal variation in bacterial disease frequency: molecular population genetic analysis of scarlet fever epidemics in Ottawa and in Eastern Germany. J Infect Dis 1993;167:759-62.
